# Application of photo-responsive metal-organic framework in cancer therapy and bioimaging

**DOI:** 10.3389/fbioe.2022.1031986

**Published:** 2022-10-21

**Authors:** Yujie Zhao, Xian Jiang, Xu Liu, Xinyu Liu, Zhihui Liu, Xiaowei Liu

**Affiliations:** ^1^ Laboratory of Integrative Medicine, Clinical Research Center for Breast, State Key Laboratory of Biotherapy, West China Hospital, Sichuan University, Chengdu, Sichuan, China; ^2^ Department of Head, Neck and Mammary Gland Oncology, Cancer Center, West China Hospital, Sichuan University, Chengdu, Sichuan, China

**Keywords:** metal-organic framework, bioimaging, photothermal therapy, photodynamic therapy, cancer therapy

## Abstract

Metal-organic frameworks (MOFs) are a class of hybrid porous crystalline materials that are assembled with metal ions/clusters and organic linkers. The fungibility of organic ligands and metal centers endow MOFs that are easy to design and synthesize. Based on their unique structure, multifarious MOFs with diverse functionalities have recently been widely applied in various research areas. Particularly striking is the application of photo-responsive MOFs in biological sensing and imaging. Notably, the photoelectronic properties make photo-responsive MOFs an ideal platform for cancer phototherapy. Moreover, ultrahigh porosities and tunable pore sizes allow MOFs to load anticancer drugs, further enhancing the antitumor efficiency. In this review, the categories and developing strategies of MOFs are briefly introduced. The application fields of MOFs in bioimaging, such as up-conversion fluorescence imaging, single/two-photon fluorescence bioimaging, magnetic resonance imaging, etc., are summarized. The working mechanism of MOFs in photo-responsive, photothermal therapy (PTT), and photodynamic therapy (PDT) are expounded. Examples of using MOFs for cancer treatment, including PTT, PDT, chemotherapy, and radiotherapy, are also demonstrated. Lastly, current limitations, challenges, and future perspectives for bioimaging and cancer treatment of MOFs are discussed. We believe that the versatile MOF will bring the dawn to the next generation of cancer treatment.

## Introduction

Metal-organic frameworks (MOFs) are a class of porous crystalline materials consisting of one-, two- or three-dimensional networks created by organic linkers and metal centers through coordination bonding (Wang et al., 2020a). Since their discovery in the late 1990s (Ding et al., 2022), MOFs have attracted great interest in various areas, including but not limited to materials science, pharmacology, chemistry, engineering, and biochemistry. Distinct MOFs can be made by combining diverse metal nodes and organic linkers. So far, many MOFs with various structures and functions have been reported.

In recent years, photo-responsive MOFs have drawn extensive attention due to their unique structural characters and photoelectronic properties (Zheng et al., 2021). Metal centers, such as Au, Mn^2+^, and Gd^3+^, endow MOFs with photoelectronic properties (Liu et al., 2022a). In addition, implanting photothermal agents or photosensitizers into their framework can produce photo-responsive MOFs. Their photoelectronic characteristics have enabled MOFs to be applied in tumor bioimaging, photothermal therapy (PTT), and photodynamic therapy (PDT). For PTT, photo-responsive MOFs generate heat under laser irradiation, exerting antitumor therapeutic effects (Liu et al., 2019). While in PDT, irradiated photo-responsive MOFs can create highly toxic reactive oxygen species (ROS) to react directly with many biomolecules in cells, thus inducing cell death and tissue lesions (Pham et al., 2021). Moreover, MOFs are efficient drug carriers because of their tunable pore size and high surface area. MOFs also have many modifiable sites that facilitate the stimuli-responsive release of loaded drugs and improve the targeting ability of nanoparticles by coupling specific polymers or tumor-targeted molecules. Therefore, MOFs are also excellent platforms for drug delivery.

Taken together, numerous studies have demonstrated that MOFs have unique advantages in bioimaging and cancer treatment. Here, we outlined the categories and developing strategies of MOFs. Meanwhile, we present the application of MOFs in bioimaging, including up-conversion fluorescence imaging, single/two-photon fluorescence bioimaging, magnetic resonance imaging, etc. Moreover, we enumerated the application of MOFs in cancer treatment, such as PTT, PDT, chemotherapy, and radiotherapy. Finally, we discuss the limitations, challenges, and future perspectives of MOFs in bioimaging and cancer treatment.

## Photo-responsive MOF

Photo-responsive MOFs have fascinating advantages of phototherapy and drug loading at the same time. In recent years, the studies of these MOFs have exhibited a rapidly growing tendency. In this section, we discussed the types and working principles of photo-responsive MOF.

### Classification of photo-responsive MOF

Based on replaceable organic ligands, metal centers, and ultrahigh porosities, MOFs not only serve as efficient phototherapeutic agents for intrinsically photo-responsive but also load phototherapeutic agents for non-intrinsic photo-responsive properties (Zheng et al., 2021). According to the type of phototherapeutic units in MOF, intrinsic photo-responsive MOF can be divided into organic-doped MOF and metal-doped MOF. Regularly arranged phototherapeutic organic and metal in the MOFs can improve photostability and bioimaging/PTT/PDT efficacy (Yin et al., 2022). Organic-doped MOFs include porphyrin-based MOFs (Begum et al., 2019; Zhang et al., 2021), bacteriochlorin-based MOFs (Zhang et al., 2019a; Pucelik et al., 2020), BODIPY-based MOFs (Wang et al., 2016; Yang et al., 2019a), Prussian blue-based MOFs (Zhang et al., 2019b), etc. Metal-doped MOFs consist of copper, iron, and manganese-based MOFs. Non-intrinsic photo-responsive MOFs do not have optical characteristics themselves. Nevertheless, their tunable structure and porosity can perform them as medicine conveyance. Phototherapeutic agents can be loaded inside the MOF or embedded in the massive pores of the MOF (Chen et al., 2015; Yang et al., 2019b; Deng et al., 2019). Moreover, some photosensitive polymers can be used as coating materials on the surface of MOFs. MOF’s high specific surface area can provide more footholds for them, bringing the advantages of the two into full play (Liu et al., 2014).

### Synthesis of photo-responsive MOF

**FIGURE 1:**
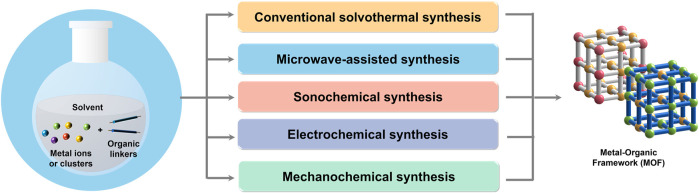
Illustration of MOF synthesis. Different methods synthesize MOF from metal ions/clusters and organic ligands, self-assembling them to form porous crystalline materials with repeating structural units.

The synthesis of MOFs is a process in which metal ions/clusters and organic ligands self-assemble to form repeating structural units (Figure 1). Their properties are determined by many factors in the progress of synthesis. Typically, these factors include the self-photoelectronic properties of the metal ions and the organic ligands, the solvent used, the reaction time and temperature, and the crystallization kinetics (Raptopoulou, 2021). In the past few decades, numerous classic synthesis methods for photo-responsive MOFs have emerged, such as hydro (solvo)-thermal, sonochemical, microwave, mechanochemical, and electrochemical methods. Among them, the hydro (solvo)-thermal method is considered one of the most popular methods to prepare photo-responsive MOFs due to its simplicity, convenience, and low cost. A kind of two-dimensional Cu-TCPP MOF with significantly enhanced photoelectric properties and dual-mode light-emitting Ln-MOFs are synthesized in this gentle way with high pressure and temperature (Xu et al., 2020; Wang et al., 2022). Moreover, selecting adequate methods, such as ultrasound (Abazari et al., 2019), microwave (Yang et al., 2022), electrochemistry (Cong et al., 2014) and mechanical force (Khosroshahi et al., 2022) to assist synthesis can shorten the reaction time and better control the particle morphology, having also been favored by scientists in recent years.

### Working principle of photo-responsive MOF

Photo-responsive MOF can be initiated to work *via* laser irradiation with a specific wavelength. Once the electrons of photo-responsive MOFs absorb photon energy, they migrate from the basic singlet state (S_0_) to the singlet excited state (S_1_). They subsequently release excess energy through various pathways, returning to the S_0_ state (Pamei and Puzari, 2019). Fluorescence emission will occur when the molecule releases its energy in the form of a fluorescent photon. This fluorescence can be used in bioimaging (Li et al., 2022).

### Working principle of MOF in PTT

For photothermal MOF materials, the working principle is complex and diverse. In most photothermal MOFs with organic dyes and polymers, their S_1_ state tends to undergo nonradiative vibrational relaxation, returning to the ground state by the collision between the chromophores and the surrounding biological environments and emitting energy as heat (Ng and Zheng, 2015). In contrast, high carrier density materials such as semiconductors, metals, metal oxides, and quantum dots can have a photothermal effect through the localized plasmon surface resonance (LSPR) (Luther et al., 2011; Long et al., 2015). When this collective oscillation of electrons decays by nonradiative transition, energy is dissipated as heat. In low electron density semiconductors, heat is generated by the relaxation of electron-hole pairs. Irradiating them will excite their electron to a higher energy status in the conduction band and leave a hole in the valence band. These electrons and holes will lose energy as heat, relaxing to the band edges through vibrational relaxation, recombining near the band edge, and further generating heat through the crystal lattice vibration (Wang et al., 2017; Li et al., 2019).

### Working principle of MOF in PDT

In PDT of photo-responsive MOF, unstable electrons of the S_1_ state will radiate some energy in the form of fluorescent quanta, subsequently converting into a more stable excited state (T_1_) (Ethirajan et al., 2011; Ng and Zheng, 2015). The photo-responsive MOF in the T_1_ state can produce cytotoxic ROS through two processes: Type I and Type II reactions (DeRosa and Crutchley, 2002; Agostinis et al., 2011). In the type I reaction, the MOF interacts directly with the cancerous substrate and creates free radicals and anion radicals through an electron or hydrogen transfer, leading to the appearance of ROS, such as hydrogen peroxide (H_2_O_2_), hydroxyl radicals (˙OH), and superoxide anion radicals (O^2−^) (Juarranz et al., 2008; Lan et al., 2019). During type II reactions, the energy of MOF in the T_1_ state is moved directly to the basic energetic state of oxygen (O_2_). Then it generates highly reactive singlet oxygen (^1^O_2_) species (Van Straten et al., 2017; Kwiatkowski et al., 2018).

## Photo-responsive MOF for bioimaging

Up to now, photo-responsive MOFshave drawn significant attention in the bioimaging field due to their advantages of acceptable biocompatibility, better dispersing property, and high biological activity (Giliopoulos et al., 2020). Numerous studies have combined MOFs and bioimaging to develop a more effective medical *in vivo* and *in vitro* imaging system (Zhu et al., 2021), including up-conversion fluorescence imaging, NIR PersL imaging, single- or two-photon fluorescence bioimaging, magnetic resonance imaging, etc (Table 1).

**TABLE 1:** The main effects and limitations of MOF.

Applications		MOF	Main effects	Limitations
Photo-responsive MOF for bioimaging	Up-conversion fluorescence imaging	NaYF_4_:Yb^3+^/Er^3+^@MIL-100(Fe) (Deng et al., 2015)	good stability; high resolution; high fluorescence efficiency	Low quantum yield
	NIR PersL imaging	PLNP@ZIF-8 (Zhao et al., 2019)	low irradiation damage; auto-fluorescence-free; deep tissue penetration	poor stability; insufficient infrared luminescence
	Single-photon fluorescence bioimaging	Hf-UiO-66 MOFs (Sk et al., 2018)	single photon sensitivity	low signal-to-noise ratio; photobleaching
	Two-photon fluorescence bioimaging	TP-MOF (Yang et al., 2019c)	high photostability; low photodamage; high spatiotemporal resolution	rely on fluorophores; cytotoxicity
	Magnetic resonance imaging	MIL-101(Fe) (Hamideh et al., 2020)	non-invasiveness; high spatial resolution; deep penetration	low signal-to-noise ratio; streak artifacts
	Computed tomography	UiO-PDT (Zhang et al., 2017)	cost-effectiveness; anatomical imaging ability; wide availability	CT artifacts
	Multimode bioimaging	ICG-CpG@MOF (Fan et al., 2021)	overcome the inherent limitations of the single-imaging technique for comprehensive diagnostic information	insufficient multimodal information fusion
Photo-responsive MOF in cancer therapy	Photothermal therapy	Cu-MOF; HKUST-1 (Yao et al., 2020)	convert light into thermal energy; non-invasiveness; safety	the limited tissue-penetration ability of light; low efficiency of photothermal conversion
	Photodynamic therapy	Au@MOF (Cai et al., 2021)	produce ROS to kill tumor cells; non-invasiveness; safety	the limited tissue-penetration ability of light; strong oxygen dependence
	Chemotherapy	Fe-TCPP MOF (Wan et al., 2019)	widely used to treat cancer; often with effective results	drug resistance of chemotherapy drugs
	Radiotherapy	MIL-100(Fe) (Du et al., 2021)	high accuracy and efficiency	inevitable recurrence and tolerance; tissue damage
	Combination therapy	PCN-224 (Li et al., 2017)	enhanced tumor treatment efficacy; reduced drug toxicity and drug resistance	difficult to control the pharmacokinetics and pharmacodynamics

### MOF based up-conversion fluorescence imaging

Up-conversion nanoparticles (UCNPs), favored by researchers for their outstanding ability to emit visible light under infrared irradiation, have been widely used in bioimaging as novel optical probes (Liu et al., 2022a). For example, using MOFs based on iron (III) carboxylate materials as the shell and β-NaYF_4_:Yb^3+^/Er^3+^ nanoparticles as the core, Deng et al. developed a novel aptamer-guided nanocarrier (Deng et al., 2015). In biological imaging applications, the UCNPs were able to emit strong green emissions for up-conversion fluorescence imaging under a 980 nm laser. Compared with the photobleaching and quenching of fluorescent organic molecules, this MOF can provide good stability, high resolution, and high fluorescence efficiency optical imaging *in vivo*.

### MOF based NIR PersL imaging

Persistent luminescence (PersL) is a phenomenon in which optical materials still emit long-lasting luminescence after absent excitation (Liu et al., 2022a). Recently, interest in the application of near-infrared (NIR) PersL nanoparticles (PLNPs) as optical probes *in vivo* bioimaging systems has increased significantly due to their biological properties such as background autofluorescence-free, high tissue penetration, and low radiation damage (Lv et al., 2019). Zhao and coworkers synthesized a persistent luminescent MOF (PLMOF) of PLNP@ZIF-8, and the continuous emission of infrared light from PLNPs in the absence of external irradiation could activate tumor site imaging without background interference (Zhao et al., 2019). Their results demonstrated that PLMOF could be an encouraging theragnostic platform for precision medicine.

### MOF based single-photon fluorescence bioimaging

Single-photon imaging can detect low-intensity light, showing weak optical signals with single photon sensitivity (Rehain et al., 2020; He et al., 2022). Mostakim and partners reported Hf-based UiO-66 MOFs with high hydrolytic stability and catalytic activity (Sk et al., 2018). No additional surface modification is required during synthesis. Due to the characteristics of rapid reactivity, high selectivity, and specific sensitivity of peroxynitrite, Hf-based UiO-66 MOFs could be applied to single-photon bioimages of living cells through the detection of intracellular peroxynitrite.

### MOF based two-photon fluorescence bioimaging

Two-photon excitation fluorescence imaging is a powerful technique for visualizing deep tissue due to its high photostability, low photodamage, and high spatiotemporal resolution (Liu et al., 2017; Lu et al., 2020). Chan Yang and his group reported a two-photon MOF (TP-MOF) based on nanoscale MOFs combined with a two-photon organic part using a click chemistry method (Yang et al., 2019c). The TP-MOF probes could ascertain and generate images in living cells and tissues within a penetration range of 130 μm due to the two-photon fluorophores excited by NIR while retaining the TP organic moiety’s fluorescence-responsive properties and possessing excellent photostability and biocompatibility.

### MOF based magnetic resonance imaging

Magnetic resonance imaging (MRI) is a leading technique in clinical imaging and pathology analysis due to its advantages in high tissue resolution, non-invasive angiography, and water imaging (Logothetis, 2008). It can detect the electromagnetic wave emitted by the applied gradient magnetic field according to different attenuations of the released energy in the various structural environments inside the material to understand the position and type of tissue to distinguish pathological changes (Slobozhanyuk et al., 2016). As MOFs are commonly used in developing *T*_2_-weighted contrast agents for MRI, the polymeric substance of MOFs and MRI can be used in complex bioimaging by providing soft‐tissue contrast without ionizing radiation (Hamideh et al., 2020).

### MOF based computed tomography

Computed tomography (CT) is one of the most crucial imaging methods in the clinic in prognosis prediction, differentiation of benign and malignant tumors, grading and staging, efficacy evaluation, and recurrence monitoring because of its cost-effectiveness, anatomical imaging ability, and wide availability (Ohlerth and Scharf, 2007). More recently, A new X-ray CT contrast agent, bismuth-NU-901 (Bi-NU-901), was solvothermally synthesized by Robison and co-workers (Robison et al., 2019). The *in vitro* studies revealed Bi-NU-901 demonstrates ∼14 times better contrast than a commercially available CT contrast agent. With the development of nano-technology, varied and adjustable MOFs can act as contrast agents to achieve better consequences and reduce toxic and adverse effects on healthy tissue.

### MOF based multimode bioimaging

Unlike the inherent limitation of the single-imaging technique, the fusion of different imaging modes, which has developed rapidly recently, could complement each other for comprehensive diagnostic information (Wu and Yang, 2017). For example, Fan et al. recently constructed a multimodal imaging nanoplatform called ICG-CpG@MOF for cancer diagnosis and treatment. This nanoplatform uses a specific MOF, MIL101(Fe), as the core carrier, then dressed with photoacoustic and fluorescent signal donors (ICG) (Fan et al., 2021). These platforms can utilize multimodal imaging for synergistic therapy, providing hopeful cancer diagnosis and treatment approaches.

## Photo-responsive MOF in cancer therapy

The synthesis convenience and structural flexibility allow guest molecules, such as nanoparticles, anticancer drugs, and biomolecules, to be implanted into the framework to produce MOFs with multiple functions, such as PTT, PDT, and tumor targeting (Cheng et al., 2022). As a protective shell, the porous crystal structure of MOFs not only has a high carrying capacity due to its large specific surface area and high porosity but also improves the catalytic activity of the nanocomponents. At the same time, their weak coordination bond ensures the biodegradability of MOF (Liu et al., 2022b). These desirable properties make MOFs promising applications in drug delivery, clinical oncology therapy, and other disease treatments (Table 1).

### Application of MOF in PTT

In PTT, by utilizing an optically absorbing MOF, light can be converted efficiently into thermal energy to kill the targeted cells in cancer and other disease treatments (Jiang et al., 2018), with non-invasiveness, safety, and efficiency in comparison with traditional oncology therapies (Zhi et al., 2020). Meanwhile, photo-responsive MOF-mediated PTT can protect normal tissues from heat damage (Liu et al., 2021; Yue and Zhao, 2021). Recently, Meizhen Yin and coworkers reported a crystalline MOF synthesized with perylenediimide (PDIs) (Lü et al., 2019). It shows high near-infrared photothermal conversion efficiency due to the stability of ambient radical anions. Unlike traditional cancer treatments, the high yield and stability endowed PDI-based 3D MOF with great potential in photothermal therapy. Moreover, the “turn-on” strategy triggered by endogenous biomarkers can simplify the composition of nanomedicines and offering hope for precision cancer treatment.

### Application of MOF in PDT

In PDT, photo-responsive MOF produces ROS, especially singlet oxygen radicals, upon exposure to light (Kwiatkowski et al., 2018). All components of the intracellular environment are affected by ROS, including but not limited to proteins and DNA, leading to necrosis or apoptosis. The fabrication of photo-responsive MOF by optimizing their size parameter can play a critical role for improving cellular response. Zhou et al. synthesized spherical TCPP-Zr-NMOF(PCN-224) of different sizes ranging from 30 to 190 nm, and then studied the size-dependent cellular uptake and ensuing PDT (Park et al., 2016). They find preferential cellular uptake of 90 nm-PCN-224 and its remarkable PDT efficacy over other sizes using HeLa cells. Cai et al. designed and constructed Au@MOF core-shell hybrids using the layer-by-layer method (Cai et al., 2021). The porphyrin ligand drives the conversion of O_2_ into ^1^O_2_ in the MOF shell upon light source stimulation. In the tumor microenvironment, H_2_O_2_ can be decomposed into O_2_ by catalytic action in the presence of the metal node Fe_3_O(OAc)_6_(H2O)^3+^ cluster of the MOF, resulting in a strong PDT effect, which promotes the tumor cell killing effects and tumor growth inhibition.

### MOF improves the efficiency of chemotherapy

MOF is becoming a leading candidate vector for drug delivery in chemotherapy due to its multiple advantages, such as adjustable pore size, variable structure, diverse functions, large capacity, and high biocompatibility (Wu and Yang, 2017; Wang et al., 2018; Wang et al., 2020b). Wan and coworkers developed a procedural release nanosystem with a Fe-TCPP MOF shell loaded with dihydroartemisinin (DHA) to treat cancer (Wan et al., 2019). The CaCO3 mineralized layer was encapsulated in the pores of the MOF to avoid drug leakage during transportation. The release of DHA from Fe-TCPP MOF and TCPP activation is triggered by high concentrations of GSH and a weakly acidic microenvironment within the tumor. The triple-combo regimen of Fe^2+^-DHA mediated chemotherapy, Ca^2+^-DHA mediated tumor therapy, and TCPP mediated PDT synthesis inhibits tumor growth and has no biological toxicity *in vivo*.

### MOF enhances the sensitivity of radiotherapy

Radiotherapy utilizes high-intensity ionizing radiation to induce DNA damage by generating ROS (Wu and Yang, 2017). However, plenty of evidence has shown that hypoxic cells are less sensitive to radiation than normoxic cells in radiotherapy. Benefiting from the porous structure, good biocompatibility, and tunable size, MOF NPs are considered ideal candidates for improving the sensitivity of radiotherapy (He et al., 2019). Du and his partners synthesized D-arginine-loaded MOF nanoparticles to avoid serious adverse effects during high-dose radiation therapy (Du et al., 2021). D-arginine attenuates tumor hypoxia by producing nitric oxide and down-regulating HIF-1α. In addition, MOF could produce free radicals to enhance the killing effect of tumor cells. *In vitro* and *in vivo* results showed that MOFs loaded with D-arginine increased tumor elimination and prevented lung metastasis in mice after radiotherapy, along with relatively low toxicity in cells and mice.

### MOF based combination therapy in combating cancer

Combination therapy has a distinct advantage over monotherapies in terms of enhanced tumor treatment efficacy, reduced drug toxicity, and drug resistance. Many efforts have been made to improve tumor treatment efficacy through multimodal combination therapies. The excellent drug carrier ability and photoelectronic properties of MOFs make them an ideal platform for coordinating cancer immunotherapy, targeted therapy, chemotherapy, and PTT/PDT. Recently, Dr. Tian and his colleagues fabricated a kind of tumor cell membrane modified hollow porphyrinic MOF nanoparticle to co-load chemotherapy drug doxorubicin (DOX) and photosensitizer indocyanine green (ICG) (Sun et al., 2021). The multifunctional nanoparticle exhibits excellent *in vivo* imaging and mediates photodynamic-photothermal-chemotherapy combo treatment. In addition, many photo-responsive MOFs have been found to have acoustic sensitivity. They can not only perform sonodynamic therapy (SDT) (Wang et al., 2020c) while exerting optical performance but also perform photoacoustic (PA) imaging. In a recent study, for example, Han and coworkers developed a kind of multifunctional H-TiO_2_/C-PEG nanosheet theranostic platform (Han et al., 2022). These nanosheets can not only act as ultrasound sensitizers but also possess good photothermal and photodynamic properties. When they are applied to imaging-guided tumor therapy, they have significant advantages in the precise treatment of tumors.

## Conclusion and perspectives

The applications of photo-responsive MOFs in biomedicine have boomed in the past decades. MOFs with intrinsic light response behavior by using photo-responsive building blocks or serve as the conveyance of phototherapeutic agents, owing to their tunable structure. The regular array of MOFs prevents aggregation and self-quenching of photosensitive units, greatly enhancing their effectiveness for phototherapy and bioimaging. MOF’s porous structure and easily modifiable properties make it a superior platform for imaging-guided therapy and combination therapies.

However, MOFs still have some limitations in cancer bioimaging and phototherapy (Zhao et al., 2022a). Firstly, these photo-responsive MOFs are still in the primary research stage, and the FDA has yet to approve them. Issues related to the biosafety of materials must be addressed if MOFs are to be applied in the clinical setting. It is necessary to optimize surface functionalization to achieve prolonged blood circulation and enhanced tumor targeting. Meanwhile, MOFs’ biodegradation and rapid *in vivo* clearance must be considered (Wang et al., 2021), evaluating their absorption-distribution-metabolism-excretion (ADME) to understand their toxicity profiles. Moreover, the tissue-penetration ability of light is limited (Chitgupi et al., 2017). MOFs with longer wavelengths, such as NIR-II, UCNPs, and two-photon activated MOFs, should be developed to improve the efficiency of deep tumor imaging and therapy. In addition, dynamic treatments usually have strong oxygen dependence (Xie et al., 2021). Therefore, increasing the oxygen delivery or combining hypoxia-activating drugs may be effective methods to overcome hypoxia in the deep tumor sites. And MOF happens to be an excellent carrier for the delivery of a variety of medicines and gases. We believe that MOF can break through the bottleneck of current conventional tumor treatments such as surgery, neoadjuvant chemoradiotherapy, and radiofrequency ablation (Yang et al., 2021; Zhao et al., 2022b). Undoubtedly, photo-responsive MOFs will be a promising area for future research and will become an essential part of medical treatment.
